# A Temperament-Attachment-Mentalization-Based (TAM) Theory of Personality and Its Disorders

**DOI:** 10.3389/fpsyg.2019.00518

**Published:** 2019-03-22

**Authors:** Sigmund W. Karterud, Mickey T. Kongerslev

**Affiliations:** ^1^ The Norwegian Institute for Mentalizing, Oslo, Norway; ^2^ Centre of Excellence on Personality Disorder, Psychiatric Research Unit, Region Zealand Psychiatry, Slagelse, Denmark; ^3^ Department of Psychology, University of Southern Denmark, Odense, Denmark

**Keywords:** personality, personality disorders, personality theory, temperament, primary emotions, attachment, mentalizing, self-consciousness

## Abstract

Theories of personality and its disorders need, from time to time, to be revised and updated according to new empirical and conceptual developments. Such development has taken place in the realms of affective neuroscience, evolution, and social cognition. In this article, we outline a new personality theory, which claims that phenomena we usually ascribe to the concept personality are best understood by postulating a web consisting of three major constituents: temperament (mainly primary emotions), attachment, and self-consciousness (mentalizing). We describe these constituents, their neurobiological underpinnings, the subjective experiences they evoke, and their behavioral implications. We discuss the relevance of the espoused theory in the field of personality disorders with references to borderline, narcissistic, and avoidant personality disorders as well as the DSM-5 alternative model. Implications for social psychology, psychotherapy, and common sense self-understanding are outlined. The theory aims to bridge previous contradictions between natural sciences and hermeneutics by its propositions of the evolution of self-consciousness.

## Introduction

What a piece of work is man, how noble in reason, how infinite in faculties, in form and moving, how express and admirable, in action how like an angel, in apprehension how like a god: The beauty of the world; the paragon of animals; and yet to me, what is this quintessence of dust?

– Shakespeare, Hamlet, II. 2

This article concerns the concept of personality, its disorders, and the phenomena it claims to cover. We will provide a contemporary definition. In the first part, we outline the content of the major constituents of personality, which can be organized according to a temperament-attachment-mentalizing (TAM) theory. The theory aims to encompass normal as well as pathological phenomena of personality. In the second part, we discuss the implications of the TAM theory as well as its explanatory power for understanding personality disorders.

The historical and conceptual background is that current theories of personality and its disorders harbor significant limitations in veridicality, scope, and comprehensiveness. This became an acute problem when the former diagnostic systems DSM-IV and ICD-10 were ripe for revision. The revision processes revealed a widespread consensus that (1) a categorical conceptualization of personality disorders was scientifically untenable and (2) personality and its disorders were dimensionally related phenomena. However, the field lacked a comprehensive unifying theory for personality and its disorders. Consequently, our American colleagues could not agree on a revised conceptualization for the DSM-5 due to unresolved theoretical and scientific issues, while WHO chose to follow a very pragmatic course with ICD-11 with few references to any underlying theory of personality except for a trait model. A trait model was also made explicit in the DSM-5 alternative model, while the level of personality functioning contained additional references to self-psychology, mentalization and psychodynamic theories, and emotion dysregulation. However, there was no theory that explicitly linked trait aspects with social cognition, self, and relational competencies.

In this article, we suggest a theoretical approach that makes such an integration possible. We would like to emphasize that this requires a theory of personality that is broad and comprehensive, moving beyond the more limited approach that considers personality as a question of individual differences in cognition, affects, and behavior. Our theoretical position has developed in dialog with important contemporary theories of personality (e.g., trait, psychoanalytic, and social-cognitive theories). We have borrowed extensively from them but will also argue that they miss crucial concepts needed for a modern integration of the rich diversity of new relevant scientific knowledge. The main aim of this article is thus to suggest a new framework for thinking about personality and for organizing the wealth of accumulated data with a special reference to the tension between contemporary personality and personality disorder discourse.

We should remember that the academic discipline of psychology in general, and personality and social psychology specifically, arose to address and ideally answer the very intricate yet also basic questions such as “Who am I?” “How does personality work?” “How and why people feel, think, and act as they do in the world?” (Allport, 1937; Robinson, 1976; Magnusson, 1999; Mayer, 2005). Such fundamental questions are essentially existential and concern the very nature and meaning of our being and existence as such – thereby reflecting humankind’s perpetual quest for self-understanding throughout our species’ intellectual history, be it in religion, art, and literature, philosophy, or science (Yalom, 1980; Kierkegaard, 1989; Taylor, 1992; Jackson, 2003; McDougall, 2003; Millon, 2012). Though the reader will of course be aware of this fact that personality and social psychology’s most basic subject matter concerns the very nature of our (mental) lives, our essence, and existence, we feel it is necessary to remind ourselves about it from the outset of this article, considering the field’s current state of affairs.

Indeed, contemporary academic psychology and allied disciplines have progressed remarkably in terms of methodology and empirical studies, as well as specialization into subfields – e.g., cognitive, developmental, personality, social, work and organizational, and clinical psychology – which also have their own journals, societies, and congresses. To be sure, this development is not only natural but also positive. As a science matures, it must diversify and specialize. These diversification and specialization, however, come with a tradeoff. It runs the risk of creating insulated and fragmented detail-knowledge, within disciplines and sub-disciplines, who becomes ever more estranged from each other, and sometimes even outright antagonistic (Mischel and Shoda, 1998; Millon, 2012; Casadevall and Fang, 2014). This state of affairs creates among other things a need for theory – a theory based on a remembrance of our common subject, the subject of human subjectivity and behavior, or personality as such, neither more nor less. We need (meta-)theory to put the pieces of specialized knowledge back together and make sense of the plentitude of empirical studies within the various subdisciplines, as well as of the findings from other scientific disciplines (Millon, 1990; Cosmides et al., 1992; Magnusson, 1999; Katzenelson, 2001; McAdams and Pals, 2006).

In the following, we will situate the science of personality among the life sciences. Personality is both nature and culture. It depends not only on genes and the brain, but also on lived experiences, subjectivity, texts, discourse, and interpretations. The fact that more species than *Homo sapiens* rightfully can be ascribed the agency of personality put evolution at the center stage. Evolutionary knowledge is crucial for a modern theory of personality. However, human personality contains something more than what it shares with, e.g., other primates. This other “thing” is above all the human capacity for self-consciousness. A theory of human personality needs to account for this extraordinary agency, self-consciousness, i.e., how nature becomes culture and vice versa, or to be more precise, the dialectics of nature and culture. As we will explain in the following, we interpolate human attachment relationships as the mediating factor in this dialectic interplay. The challenge is to integrate evolutionary determinants with lived experiences and cultural artifacts as means for understanding minds, in one coherent theory.

## Personality – What is it?

Personality is a commonsense concept. People use the noun as a matter of fact, and everybody appears to “know” what personality is in everyday life. The construct is part of folk psychology (Fiske et al., 2007; Heine and Buchtel, 2009) and is used both implicitly and explicitly by laypersons to understand and make sense of oneself and others (Dweck et al., 1995; Srivastava et al., 2010). When common sense moves to science, however, problems arise since personality then reveals its nature as a psychological construct. Personality is not a delineated thing – not a *res extensa* in a Cartesian sense. It is not like body organs, such as the heart which can be dissected and studied under the microscope. In contrast to the heart, personality as a word and construct does not have a concrete designata to which we can point (define it ostensively) and say “look, this is personality.” Personality is essentially no-thing but that does not make it nothing, to paraphrase Katzenelson (1989). Personality does not exist, nor can it be described and understood in its own right, but only through its relation to something else. In scientific terms, the constituents of personality are latent variables that dispose each and every specific person, to think, feel, and (re-)act as they do, in their very own characteristic ways as they move in and through the world (Borsboom et al., 2003).

How might we then define personality? Inspired by prominent scholars within the field (e.g., Bronfenbrenner, 1981; Millon, 1990; Buss, 1998; Mischel and Shoda, 1998; Livesley, 2003; Cacioppo, 2004; McAdams and Pals, 2006), our position on the requirements to a definition of personality is this:

1. The definition should be broad, comprehensive and include references to the nature of man, and use words and expressions that refer to established theoretical traditions that are empirically grounded in modern science (e.g., evolution, neuroscience, and sociology) (Millon, 1990; Cosmides et al., 1992; Buss, 2001; Gottlieb, 2007). The scientific basis of the theory should encompass natural sciences, life sciences, psychology, hermeneutics, and philosophy. That is, explanatory principles should include brain processes, development, intersubjectivity, and sociocultural processes. We would like to emphasize that because life sciences are very much involved, the theory should explicitly refer to evolution, acknowledging from the outset that personality concerns other creatures as well as human beings.
2. The definition should have as its referential source a comprehensive theory in which the different explanatory principles should be linked to each other, conceptually as well as empirically. That is, the one should build upon the other. For example, emotions, attachment, and self-consciousness should be linked in an intrinsic way.
3. The crucial elements of the constitutive components should be measurable.
4. The explanatory power of the theory should be large. It should provide directions to search for answers to existential questions like “who am I?” for ordinary people, as well as being relevant for research on personality and its disorders through making sense of the empirical literature across sciences and scientific disciplines, as well as being heuristic in generating empirically testable hypothesis for future research.
5. The theory must not only bring together various sciences and subdisciplines within academia but certainly also bridge the artificial divide between so-called normal versus abnormal personality, as research suggests that these concepts lie on the same continuum (Markon et al., 2005; Oltmanns et al., 2018).

According to the principles discussed above, and inspired by the work of McAdams and Pals (2006), we propose the following definition of the concept personality:

Personality is the unique variation in the individual of the evolutionary-based foundation of human nature, as well as of attachment and self-reflective abilities. Personality is expressed as developing patterns of dispositional traits, characteristic adaptations, interpersonal relations and integrative life-stories that are complex and interwoven in cultural matrices and interpersonal contexts.

This text defines personality with references to three major fields of life science and psychology: (1) evolution (Darwin, 2011), (2) attachment (Bowlby, 1997), and (3) the self (Fonagy et al., 2007; Kohut, 2009). Furthermore, it has references to personality traits (Allport, 1937), adaptations (Buss, 1998), interpersonal relations (Bowlby, 1997), narrative theory (McAdams, 2018), and psychodynamic theory and group analysis (Foulkes, 1990).

## The Three Major Constituents of Personality

We will argue that a modern and integrative conceptualization of personality calls for three major constituents: *temperament, attachment, and mentalizing*. These constituents come in the following evolutionary order and build upon each other: first comes temperament, which is a prerequisite for attachment, which in turn is a prerequisite for mentalizing. Furthermore, we will argue that the elements of temperament have undergone natural selection according to established evolutionary principles. Attachment has some general features that link it to temperament, but the prototypal attachment style of the individual is mainly based on lived experience. Mentalizing is an ability, which develops within the context of attachment relationships and also entails the internalization of cultural achievements and codes. There is thus a movement in historical times, and in the ontogenesis of the individual subject, from nature (evolution and phylogenesis) to intersubjective learning, symbolization, and cultural internalization (socio-cultural processes). Individuals are coined in different and distinctive ways by these processes. That is what we label personality.

### Temperament

We chose to label the most hereditary and evolution-based constituent of personality as temperament to honor the specific tradition of Western thinking since Hippocrates. This is a rather conventional position. Most personality theorists adhere to a temperament or hereditary component of personality (Millon, 1996; Cloninger, 2008; Kernberg, 2016). Of note, temperament has also been used within the literature of child development to denote the individual characteristics of infants and children, leading to a state of conceptual confusion in the field making it difficult to synthesize findings on infant temperament with personality research (De Pauw and Mervielde, 2010; Shiner et al., 2012). Despite differences in definitions of temperament, most authors generally posit mood or emotions and emotion regulation at the heart of temperament (Stanton and Watson, 2014; Karterud et al., 2016). We share this position, and to conserve space, we shall, in this article, downplay the executive components of temperament (e.g., attention) and focus primarily on emotions. Yet, we will also add two components that are not emotions proper, i.e., social rank behavior and conscientiousness.

Concerning emotions, we will distinguish primary (or basic) emotions from complex/social emotions. This does not mean that primary emotions are not “social,” but by convention higher order and more, complex emotions are typically defined as social (Ekman, 1992; Hareli and Parkinson, 2008). There is some disagreement about how many and which emotions deserve the label “primary.” Prominent authors are Tomkins (1981), Ekman (1992), Damasio (1996), Darwin (1999), Plutchik (2000), Panksepp (2004), Izard (2007), and Panksepp and Biven (2012). Of these, we consider Panksepp as currently having the most thorough scientific grounding for his theory (Watt, 2017). Panksepp was truly an experimental researcher in neuroaffective science and developed a most stringent definition of what should count as “primary emotions.” By definition, primary emotional systems should:

1. Be found among all mammals,
2. Represent adaptive functions with respect to life-threatening challenges,
3. Be accompanied by typical behavioral patterns,
4. Be accompanied by typical physiological reactions,
5. Have an anatomical localization in the brain implying that they can be activated by site-specific electric stimulation, and
6. Be dependent on specified hormones, neuropeptides, and neurotransmitters, implying that they can be manipulated by biochemical substances.

According to these criteria, Panksepp maintained that there exist seven primary emotions, labeled (with bold characters according to Panksepp’s style): SEEKING/anticipatory joy, FEAR, RAGE/anger, SEXUAL LUST, CARE/love, SEPARATION DISTRESS/sadness, and PLAY/joy. All primary emotions are “relational.” Their purpose is to regulate the organism’s relation toward other creatures in the living world. Some emotions even predate the mammals, e.g., fear and rage, which can also be found among reptiles. People vary and differ in their respective intensity and proclivity for the various primary emotions (Karterud et al., 2016; Montag et al., 2016, 2017; Montag and Panksepp, 2017). There is no space to describe the primary emotional systems in detail. We restrict ourselves to some clarifying comments, following Panksepp (2004); Panksepp and Biven (2012).

SEEKING is the most basic emotion – the prime mover. It is an all-purpose system that “drives” other systems, like sexual lust and play. Other labels of this system, found in the literature, are exploratory behavior (Buchholtz and Persch, 1994), behavioral activation system (Pickering and Gray, 1999), novelty seeking (Cloninger et al., 1993), libido (Freud, 2017), or the reward system of the brain (Berridge and Kringelbach, 2015). Seeking is turned on when we wake in the morning and orient ourselves toward the surrounding family and job obligations or when we look forward to the football match of the night when we can scream and behave playful in a childish manner. Seeking is low-keyed in depression and up-tuned in manic states. Seeking is mainly driven by the neurotransmitter dopamine as attested by people going at rave parties the whole night by taking dopamine agonists like amphetamine. The subjective feeling of being aroused by seeking is *anticipatory joy.* It is worthwhile enduring much hardship if we can look forward to meeting a loved one, a gourmet meal, sex, recognition, an opera performance, or reading a longed-for book. People are different with respect to their activity level and curiosity – basically their SEEKING.

FEAR is a most unpleasant feeling, unlike seeking, and it is involved in negative reinforcement. Fear makes us avoid things. There are a few unconditioned stimuli that evoke fear among humans, among them is pain. However, *Homo sapiens* can learn to fear almost everything. Amygdala and periaqueductal gray (PAG) are trigger sites for fear, and the neurotransmitter glutamate is involved. Fear can be reduced by chemicals that affect the GABA transmitter system, e.g., benzodiazepines. When the source for fear arousal is known, we usually label the accompanying feeling simply as fear (e.g., fear of flying). When the source is unknown, we label it anxiety.

SEXUAL LUST is of course a primary emotion. It is definitely a motivator of outmost importance for the survival of the species and hence a part of personality. People differ grossly in their threshold for sexual activation and sexual conduct, due to an interplay of sex hormone levels, learned habits, and different moral reasoning. However, differences among individuals in sexual lust, and their implications, remain understudied in research of personality and its disorders, as well as in psychopathology more broadly (Forbes et al., 2017; Kashdan et al., 2018) – despite the historical legacy of C. Darwin and S. Freud.

CARE is a prerequisite for attachment behavior. Being aroused by care is accompanied by feelings of love, empathy, fondness, belongingness, wanting to take care of, etc. CARE is promoted by hormones and neuropeptides such as endogenous opioids and oxytocin, so-called prosocial agents. Endogenous opioids and oxytocin make you relax and calm down, eventually experience moments of bliss and peaceful happiness. Individuals with, e.g., antisocial personality disorder have high threshold for care and consequently trouble with calming down and obtaining experiences of internal warmth and peace. Consequently, they turn more often than other personalities to soothing chemicals as external opioids.

SEPARATION DISTRESS is closely connected to care, and both of these primary emotions are crucial for attachment. Separation distress can be intensely unpleasant, amounting to despair and bottomless sadness with loss of hope for the future. The separation distress system is involved in grief and depression (Panksepp and Biven, 2012). The ultimate or ideal “cure” for separation distress is being reunited with the loved one. The experience of being met, reunited, loved, and cared for increasing the endogenic opioids and alleviating the panic of being lost forever. Individuals with borderline features have a particularly low threshold for separation distress, expressed in the diagnostic criterion of desperate attempts at avoiding abandonment. The hypersensitivity of borderline patients for separation distress is probably underestimated (Gunderson and Lyons-Ruth, 2008). They seem to need more than usual doses of affirmation, mirroring, inclusion, etc. in order to feel a cohesive sense of self.

RAGE is also necessary for survival. It is not conceived of as a drive, as in psychoanalysis, but as an emotion, which is triggered by certain situations, most typically when being threatened or humiliated, being blocked by other persons in obtaining valuable goods (seeking) or when ruminating on revenge due to earlier humiliations. Rage can be provoked by electrical stimulation of the PAG brain region. Animals then display the typical rage expressions of showing teeth’s, flattening their ears, narrowing their eyes, wrinkling their eyebrows and nose, attacking lab personnel, etc. As we all know, people are very different with respect to thresholds for rage. Some individuals never experience rage, while others go around as ticking bombs.

PLAY can also be observed among all mammals, e.g., as rough and tumble play. It is believed to serve socializing functions, particularly by taming rage and learning basic skills and cultural norms. It is of outmost importance for animals (and humans) to know when “enough is enough,” when to signal subordination or victory, and avoid being “unnecessarily” hurt. Thus, mammal fighting has an inborn ritual or pretend mode character. One should stop at certain levels. Primates that do not learn the local rules for the troop tend to become outcasts. Infants can be observed to be engaged in rough and tumble play almost endlessly, and it is obviously accompanied by subjective experiences of joy. Notwithstanding the positive psychology movement (Seligman and Csikszentmihalyi, 2000), play and joy (which is the subjective experience of being aroused by play) are underestimated by most modern psychological theories, notably the psychopathological ones (but for exceptions, see e.g., Watson and Naragon-Gainey, 2010; Stanton and Watson, 2014), especially considering the large body of evidence linking positive emotions to mental health (Fredrickson, 2013). This is most peculiar since people (in the Western world at least) are intensely engaged in play and joy during most of their leisure times, being it of the more passive kind of looking at entertainment TV programs or by hobbies and sports. And, most important, people are very different with respect to play. Some are very serious and almost never engage in proper play, while others are truly exemplars of Homo Ludens.

### Other Temperamental Dispositions

There are other temperamental dispositions that are not primary emotions in a classical sense. Foremost of these are *social dominance* and *conscientiousness*. Social dominance (or social rank behavior) is a component in several temperament constructs, e.g., the interpersonal circumplex (Stevens and Price, 2000; Fournier et al., 2012; Johnson et al., 2012; Hopwood et al., 2013; Stanton et al., 2017). It is an observed and separate temperamental disposition among most social animals and individuals differ with respect to their inclination (van der Westhuizen and Solms, 2015; Qu et al., 2017). Not everybody competes for the alpha-male/female position, and some subordinate more easily than others (Sloman and Gilbert, 2010). Among humans, it is a most potent motivational force. Since the dawn of time, *Homo sapiens* have been fighting for power, status, and wealth. Males are known to do so more frequently than females, and the faculty of social dominance is found to be linked to sex hormones, such as testosterone (van der Westhuizen and Solms, 2015). Social dominance is also a prominent feature of narcissism, while anxious/evasive personalities are inclined to favor subordinate positions (Karterud, 2017).

Conscientiousness is another well-researched temperamental disposition, which is included in the five-factor model and describes the tendency to be organized, self-controlled, delaying gratification, hardworking, following rules and norms, planning ahead, and being perfectionistic (Roberts et al., 2009; Stoeber et al., 2009; Fayard et al., 2012; Eisenberg et al., 2014). Individuals differ highly with respect to this inclination. Some are orderly and perfectionistic, while others create a mess wherever they go. One might speculate about the evolutionary origin of conscientiousness. What purpose does it serve? In modern life, it might seem obvious that conscientiousness is a valued characteristic, which underlies socially functional and acceptable behavior (Boyd and Richerson, 2009; Miller, 2009; McCabe and Fleeson, 2016). But what about its phylogenesis? One evolutionary source might be hoarding behavior, which becomes important when animals tend to occupy permanent residences, such as nests (Hummelen et al., 2008). However, perfectionism is difficult to observe among animals, and another possibility is that this trait tendency has a rather late evolutionary history, e.g., that it develops by the species *Homo* who came to rely on tool-making practices (which is almost absent among other species). Conscientiousness is a sine qua non for modern technology-based societies. The more specialized a society, the more important is conscientiousness and perfectionism. If the tools are not exact in the smallest details, the watches and time control would fail, the internet would fail, medical diagnoses would be incorrect, atom bombs might explode, etc. Moreover, modern western humans engage in world championships all over. Fueled with social rank behavior, humans train to achieve perfectionism in almost everything, be it violin playing, chess, football, science, dance, shooting, fishing, singing, hair dressing, etc. Hence, conscientiousness has grown increasingly important throughout the anthropo- and sociogenesis of our species, facilitating the building of complex and sophisticated tools, cultures, and modern societies (Tomasello and Vaish, 2013; Hare, 2017). Moreover, conscientiousness *in extremis* is linked to obsessive-compulsive personality disorder and personality pathology in the DSM-5 Section III alternative model as well as the ICD-11 in the form of rigid perfectionism and perseverance and anankastia, respectively (Bach et al., 2017).

### Attachment

In an evolutionary sense, attachment is a relatively new reproductive strategy that developed some 150 million years ago with the mammals, whereby a limited number of offspring were taken good care of, instead of a large number that were left to their own destiny. Attachment depends on the offspring giving distress signals/calls when in need or in danger (and thus being aroused by FEAR or SEPARATION DISTRESS), and the mother/parental figure responding with CARE. As these interpersonal transactions are successfully repeated throughout development, in normal instances, there develops a positively loaded emotional bond between the agents (Bowlby, 1997). There is a crucial difference between temperament and attachment. Although temperament can be modified through a civilizing process, it is there, from the very outset, as behavioral dispositions. Attachment is potentiated by the primary emotions of fear, separation distress, and care, but *the behavioral pattern that develops in the individual (child) is primarily something learned through experience*, based on successive intersubjective interactions with its caretakers and social surroundings. Thus, the genetic loading of attachment behavior is only modest (Fraley et al., 2013; Mikulincer and Shaver, 2018). There is apparently no specific gene(s) for attachment, whereas we consider the components of temperament to be linked to endophenotypes (see Panksepp, 2006).

Based on (early) attachment experiences, Bowlby (1998a,b) claimed that the child constructs internal working models of living creatures in the world, above all of human beings. And that these models were some kind of replicas of parental figures that came to represent the basis for later interpersonal relations. While temperament regulates the relation to other living organisms with respect to important life domains, it is not until attachment becomes rather sophisticated that individuals come to form *internal representations* of other living creatures. This capacity is present to some extent among other primates. Among chimpanzees, the individual members of the group (of say 50–70 members) usually know the characteristics, bloodline, and social rank of all other members. When baboons are used as sheep shepherds, they are said to know individual characteristics and family relatedness of a large number of sheep (Cheney and Seyfarth, 2007). Thus, it is plausible that some animals have internal representations of other social agents, but the quality or mental sophistication of these representations (or working models) is likely rather general compared to those in humans (Call and Tomasello, 2008).

Early attachment experiences lead to the development of typical attachment orientations, e.g., secure and insecure (overinvolved, dismissive, or disorganized) (Bretherton, 1992; Bo et al., 2017). These patterns are powerful organizers of intimate relationships and influence adult interpersonal behavior to a large degree. However, one might argue that the attachment tradition overestimates its scientific accuracy by defining categories of attachment patterns. Most probably, they are dimensional in nature, as is the internal working models (Bartholomew and Shaver, 1998; Rutter et al., 2009; Beeney et al., 2017). In real life, people are more or less secure (or insecure), more or less overinvolved, or dismissive. Nevertheless, people are characterized by strong interpersonal dispositions, which rightly qualify as personality characteristics, though they may vary throughout the lifespan (Shaver and Mikulincer, 2005; Chopik et al., 2013). And insecure attachment, particularly of the disorganized type, appears to play an important role in psychopathology in general and the development of personality disorder specifically (Lyons-Ruth et al., 2006; Cheney and Seyfarth, 2007; Mikulincer and Shaver, 2012). The phenomenology of disorganized attachment will become apparent in group situations, e.g., psychotherapy groups (Morken et al., 2014). We are talking about individuals that often end up in outsider positions, who are socially clumsy and do not adapt readily to the prevailing social norms, who seem to lack strategies for engaging in close and intimate relationships, and who are confused about own needs and motivations and may communicate this in highly maladaptive ways.

The crucial point for defining attachment as a personality constituent is that individuals are highly different in this respect and that these differences have huge consequences for their life trajectory, including emotion regulation, interpersonal functioning, and well-being (Reizer et al., 2013; Bo and Kongerslev, 2017; Mikulincer and Shaver, 2018).

While Bowlby’s theory was initially met with doubts and devaluation among the British psychoanalytic community, the attachment theory in our days is almost paradigmatic. It has influenced most schools of psychology and psychotherapy and has had a tremendous impact on Western thinking on mother/father-child relations, child welfare programs, kindergarten politics, family politics, etc. (Smith et al., 2017). Bowlby was right in emphasizing the survival value of attachment, but newer theoretical developments suggest that attachment relations in early development are not only important in terms of physical survival, but also for fostering the capacity to mentalize and engage in trusting relations to others – which is vital capacities for understanding oneself and others, moral behavior, and hence for becoming a competent actor and participant in human social relations and society at large (Fonagy et al., 2011; Baumeister and Vonasch, 2012).

### Consciousness

Before considering mentalizing, we have to take a detour by consciousness because mentalizing is intimately linked to our species’ unique capacity for self-consciousness and reflection. However, consciousness has an older (both evolutionary and ontogenetic) manifestation, which is *core consciousness* (Panksepp, 2004). Much confusion in the discourse on consciousness arises when these two manifestations (core versus self-consciousness) are not distinguished. The phenomenon of core consciousness has different names (e.g., anoetic consciousness). It concerns *consciousness of the world* through the senses (sight, hearing, smell, taste, and touch) whereby the world lightens up and becomes more manageable. Feinberg and Mallatt (2016) convincingly argue that this evolutionary progress was dependent on a certain complexity of brain circuits and that it occurred around 550 million years ago. They suggest that this development also includes (proprioceptive) consciousness of bodily posture in space as well as *(raw) emotional experience.* This has been a most controversial topic, i.e., *if animals feel their emotions*. Is my dog conscious of the obvious joy it seems to harbor when I return home each afternoon? Yes, today opinions have changed in favor of acknowledging emotional consciousness among higher animals. However, this does not imply that my dog (or other animals) *knows* that it is joyful. That would imply a capacity for self-reflection.

*Consciousness of the self* depends on there being a core self (e.g., agency, emotional experience, temporal coherence, and bodily demarcation) to be thought about. Such self-consciousness might be very primitive, insightful, and sophisticated, or even delusional (Parnas and Henriksen, 2014; Zandersen et al., 2018; Zandersen and Parnas, 2019). The realization that self-consciousness is not an either-or phenomenon is a fruit of modern thinking and research. It is dimensional along a non-linear axis from immaturity to maturity (Stern, 2000). It takes time to develop self-consciousness, and it is not “really there” until the age of 4–6 years (Rochat, 2003; Fonagy et al., 2005). That is when *narrative identity* proper starts to form. Before that time most children cannot organize their experiences in narratives like “this happened to me because of or concurrent to …, and thereafter I thought ….” In infancy and early childhood, memory is largely composed of scattered episodes, which may be organized, by all means, in, e.g., schemes, but not meaningfully (in a strong sense, e.g., narratively) related to each other (though they may become so later through *post hoc* reflection). Self-consciousness, and narrative identity is something that starts out in darkness, yet gradually builds up throughout childhood and adolescence (Reese et al., 2010) and may develop to an immensely rich life history and mental ability in adulthood and old age (Singer et al., 2013; McAdams, 2018), underpinning creativity and personal meaning making (Goldberg, 1993; Bohlmeijer et al., 2007). Hence, what we in everyday discourse refer to as the self is the core self being modified and integrated by self-consciousness.

### Mentalizing

Mentalizing is a prerequisite for self-consciousness. Mentalizing is by and large synonymous with social cognition, theory of mind, or metacognition (Kongerslev et al., 2015; Bateman and Fonagy, 2016), i.e., the realization that intentional agents behave according to a different kind of logic than the non-living world. Mentalizing is to understand one’s own and others’ reactions and behaviors according to underlying intentions and cultural codes (e.g., wishes, beliefs, and desires) (Bateman and Fonagy, 2016). It is to understand that there is an opaque mind inside ourselves and other people and that minds operate in certain manners – in their own psychological ways, just as matter adheres to the laws of physics. Mentalizing is per definition a praxis of interpretation (Ricoeur, 1981; Bogdan, 2000). In a history of idea perspective, its ascendance to the forefront of psychological research interest since the dawn of the twenty-first century was dependent on the philosophical tradition of hermeneutics (Heidegger, 1979; Gadamer, 2013), which reached its pinnacle with the famous work of P. Ricoeur (1992) *Oneself as Anothe*r. Here Ricoeur fulfills a theme that was already investigated by G. F. Hegel in *The Phenomenology of Spirit*, where Hegel claims that the self needs recognition from others in order to become itself. In Ricoeur’s words, it takes the detour of the world, the signs, and symbols created by the surrounding culture as personalized in the understanding by another mind, to achieve knowledge of oneself. One has to look upon oneself from the outside, “as another.” This figure of thought of course bears resemblance to the theories of Mead (1967), Vygotsky (1978), Kierkegaard (1989), Bogdan (2000), and Allen et al. (2008). Én passant it should also be mentioned, that an implication of this line of thinking, is that any psychology of human personality must be social, not (only) in an external way, e.g., through including the social context of the person, but also intrinsically, through incorporating how the social, the generalized other, to couch it in Mead’s terms, permeates the subjective life of every person to the very core (Stacey, 2016b).

Self-consciousness is explicit mentalizing turned toward the self. By that move, core self-experiences gain shape, texture, and meaning according to cultural signs, symbols, and codes. One becomes understandable to oneself. The self lightens up, so to speak, like the world is lightened up by virtue of the senses. Explicit mentalizing is a cultural achievement, although it builds on the capacity for implicit mentalizing. Implicit mentalizing is the immediate (default) pre-reflective understanding of self and others in daily life that is more or less effortless and makes our interpersonal transactions smooth and effective. Its source is probably an innate capacity to interpret other living creatures as intentional agents. We find it in other primates, e.g., chimpanzees, in a more primitive format.

Research during the last decade has revealed that though mentalizing is a fundamental capacity of our personality, it is also a difficult to obtain (develop-)mental achievement that cannot be taken for granted. Given poor socio-economic conditions with poverty, low social capital, family disruptions, violence, misuse, neglect, traumas, criminality, drug addiction, etc., the development of mentalizing abilities in children will suffer and thereby their adaptive capacity and resilience (Fonagy et al., 2007, 2017; Ungar et al., 2013; Ungar, 2015). Studies suggest that poor mentalizing abilities are significantly correlated with mental dysfunction, low adaptive functioning, and subjective distress; and in the population at large, individuals vary with respect to their level and style of mentalizing, which in turn have major consequences for their course of life (Lyons-Ruth et al., 2006; Katznelson, 2014). That is why mentalizing counts as a strong personality qualifier.

## The Intrinsic Links Between Temperament, Attachment, and Mentalizing

A fundamental requirement for a coherent theory is that the major concepts are related to each other in an “intrinsic” way, and not just added to each other because of statistical correlations or convenience. The links between primary emotions and attachment are obvious because attachment is but a specialized case of primary emotions “in action.” What is the link between primary emotions and mentalizing? Since Darwin’s (1999) publication *The Expression of the Emotions in Man and Animals*, there has been a prolific research tradition on the seemingly universal facial and postural display of primary emotions (Eibl-Eibesfeldt, 1989). The evolutionary benefit for both predator and prey, friends and enemies, seems to be that the agents “instinctively know” the intentions of the other. You do not need a University degree to understand a friendly smile or a hostile face and body posture. The universality of emotional display will thus favor the selection of precursors of implicit mentalizing. When the mother smiles at the baby in an attuned, cozy way, the baby “interprets” this as an invitation to intersubjective cuddling. If, by contrast, the mother yells at the baby with an angry face display, this evokes fear and separation distress and elicit distress calls, like crying, which signals that the baby perceives a danger. There is a large body of research that indicates that the further development of this incipient capacity for (implicit) mentalization to fully fledged explicit mentalizing depends on the quality of the attachment relationship, namely the sensitivity of the caregivers, and their ability to accurately mentalize and mirror the infant in a congruent and marked way (Fonagy et al., 2007; Ensink et al., 2016; Bo et al., 2017; Mikulincer and Shaver, 2018). Thus, primary emotions are prerequisites for attachment, which in turn is responsible for mentalizing and self-consciousness to develop, whereby civilization reproduces and renews itself. Civilized societies depend on the faculty of imagination, whereby the future can be thought about and there being a spring for art and science. Imagination stands on the shoulders of explicit mentalization (Bogdan, 2013). The main point here is that imagination proper presupposes a representational mind (i.e., that mental contents represent something). Without that cognitive capacity, which is achieved around years 4–5, the content of imagination is confused with reality (e.g., psychic equivalence thinking).

### The Fabric of Personality

The final question concerns the broad outline of the total web in which these constituents are interwoven and embedded. Modern neuroscience has to some degree mapped the localization of the crucial elements and, not at least, the neuronal circuits that connect and underpin them. Generally, the most primitive primary emotions have their organic substrate in deep subcortical areas of the brain (e.g., PAG, as a site for fear and rage), the attachment emotion of care being located more in the midbrain (so-called “limbic” structures), and social cognition being located in certain areas of the neocortex (Lieberman, 2007; Adolphs, 2009; Panksepp and Biven, 2012; Fonagy and Luyten, 2016). However, the more complex picture among adults is that emotions, interpersonal relations, and social cognition are integrated (“mentalized”). Take primary emotions. Primary emotions may be triggered as unconditioned raw emotional experience. Yet, primary emotions undergo a secondary learning process based on conditioning (where amygdala, hippocampus, and the so-called brain reward system are involved), which means that primary emotions (e.g., fear) might be triggered by a wide range of stimuli. Moreover, these stimuli become linked to internal representations of others (so-called internal working models), established in the wake of attachment relations, whereby relations to others become imbued by emotional coloring (“my uncle is a scary person which I tend to avoid, but my aunt is lovely and makes me smile”). The sentences in parentheses denote that the internal working models for the individual in question (to uncle and aunt) have been processed at a cortical, cognitive level as well as by amygdala and hippocampus. The representations at this tertiary (cortical) level make it possible to modify behavioral tendencies at the lower limbic level, e.g., finding a compromise between the tendency to avoid the uncle and to approach the aunt. There are thus both bottom-up and top-down processes. Through these “bottom-up and top-down” processes, self and object representations become more differentiated and colored as well by more differentiated self-conscious emotions (e.g., shame and guilt). Self-conscious emotions thus come to play an important mediating role for social norms and standards being internalized into the fabric of personality, as outlined for example by Zinck (2008). Different schools of psychotherapy situate themselves in different positions in relation to these two-way processes. For example, emotion-focused therapies favor the subcortical levels and behavioral therapies address the conditioning processes in the mid-brain, while cognitive therapy favor the cortical representations. The TAM theory of personality is thus a way of organizing knowledge that yields meaning to a wide range of psychological and psychotherapeutic schools.

## Discussion and Implications

So far, we have outlined what we consider as the three major constituents of personality through integrating knowledge from evolutionary theory, neuroscience, developmental psychology, philosophy of mind, psychopathology, and personality and social psychology. In this final section, we turn to the implications of TAM theory for understanding personality disorders, as well as for self-understanding more generally, and social psychology. We begin with a brief discussion of TAM theory and its relationship to other personality theories. Due to space limitations, we restrict ourselves to three of the most influential theories: the five factor model, social-cognitive personality theory, and psychoanalysis.

### Comparison of the TAM Theory to Other Major Personality Approaches

#### The Five Factor Model (FFM)

The FFM (McCrae and Costa, 2003) claims that personality is composed of the following five factors: neuroticism, extraversion, openness to experience, agreeableness, and conscientiousness. The model has impressive empirical support, but, strictly speaking, as the label makes explicit, it is not a theory in a comprehensive sense, but an empirical model. FFM is derived from statistical calculations based on the original idea of a “lexical hypothesis,” espousing that language will invent words and expressions that correspond to personality differences between people (Block, 1995). As such, it is a “top-down” strategy, as opposed to the theory of Panksepp’s primary emotions, which can be considered “bottom-up,” since it is based on extensive animal studies. The strength of the FFM is its measurability, reliability, and predictive validity, and it has attained high popularity in diverse sectors of psychology, ranging from psychopathology to work recruitment (Roberts et al., 2007; Kotov et al., 2010). Furthermore, the TAM theory is in depth to FFM because it has “borrowed” the FFM factor of conscientiousness.

The limitations of FFM becomes obvious when we consider it from the perspective of a comprehensive theory of personality (which, to be fair, it was not meant to be, from the outset). The coverage is limited. The FFM has little to say with respect to personality development, consciousness, identity, and the self. It is more so that the FFM *presupposes a self*, rather than it can explain the self. The construct validity of the factors is also questionable. For example, the factor neuroticism is somewhat dubious since it lumps together as diverse primary emotions as fear, rage, and sadness. So, what is the essence of the FFM factors? They are apparently not endophenotypes. Proponents of the FFM suggest that one has to go to the facet level (which separates the primary emotions) in order to obtain a good explanatory power for the personality disorders (Widiger et al., 2013). To be sure, the FFM is considered to represent temperamental aspects of personality and can accordingly demonstrate relatively high hereditary loadings (Power and Pluess, 2015). However, the factors of FFM also correlate significantly with primary emotions (Montag and Panksepp, 2017). Adding several cohorts from different countries, neuroticism correlates in the range of 0.75 with FEAR, 0.65 with SEPARATION DISTRESS, and 0.45 with RAGE. Extraversion correlates in the range of 0.55 with PLAY and 0.40 with SEEKING. Openness for experience correlates in the range of 0.40 with SEEKING, and agreeableness correlates in the range of 0.45 with CARE and negatively 0.40 with RAGE. It is therefore a plausible hypothesis that the factors of FFM obtain much of their evolutionary, biological, and hereditary significance from being a kind of proxy operationalization of primary emotions. That would also provide an explanation for why the FFM seems useful for capturing animal personality characteristics (King and Figueredo, 1997).

#### Social-Cognitive Theory and Psychoanalysis

While trait models have been highly influential within the field of personality research, another major approach has been social-cognitive, which focuses more on the intra-individual dynamics of personality (Cantor, 1990; Mischel and Shoda, 1994). This approach tends to focus less on how personality can be described (which belongs to realm of traits and trait structure) and more on how it is expressed in terms of causal structures and functions (see e.g., Mischel and Shoda, 1998; Caprara and Cervone, 2000; Hopwood, 2018). This broad perspective on personality is captured within the TAM model in terms of attachment and mentalizing. Here the more dynamic, idiographic, and inter-personal aspects of personality functioning are captured, including internal working models of self and others, and their development and importance for behavior in terms of guiding the persons interpretations of self, others, and situations. In other words, whereas trait approaches, like the FFM, have their strengths as nomothetic descriptive models, social-cognitive theories focus more on dynamic and idiographic aspects of personality.

Historically, Freud was one of the first to endeavor a social-cognitive oriented account of human personality, based on depth hermeneutical studies of the individual with a special emphasis on unconscious processes of the mind. Whereas the inventors of FFM relied on the lexical hypothesis and factor analysis, psychoanalysis relied on intersubjective competence and interpretational expertise. Psychoanalysis today is a broad family of related theories and practices, and currently, there exists no one canonized psychoanalytical personality theory (Wallerstein, 1992). However, since the “relational turn” of psychoanalysis around the beginning of the twenty-first century (Beebe and Lachmann, 2003), we might speak about a kind of current “mainstream psychoanalysis,” which contains some fundamental personality theory assertions. Since Freud, there has been a development from an emphasis of unconscious versus conscious conflicts (pleasure versus reality principle), over structural conflicts (Id, Ego, and Superego), to relational needs and conflicts. The strength of psychoanalytic personality theory is its persistent aspiration of trying to account for the dialectics of subjectivity in terms of unconscious dynamics of relational needs and conflicts, how they are handled in the unconscious layers of the mind by defense mechanisms and how they are played out in current relationships, including the transference to the therapist. Bowlby, above all, alerted us to the significance of the attachment bonds, separation anxiety, and internal working models of the mind. The TAM theory is very much influenced by this tradition and the unconscious mental mechanisms described by psychoanalysis, but in a modified version (see e.g., Kihlstrom, 1999; Stacey, 2016a; Bargh, 2018). Panksepp’s theory of primary emotions has influenced psychoanalysis, particularly those of a neuropsychoanalytic orientation (e.g., Zellner et al., 2011), and one of his last books was written in collaboration with a psychoanalyst (Panksepp and Biven, 2012). That said, it will not be correct to state that the primary emotion theory is widely embraced in contemporary psychoanalysis, nor the theories of attachment and mentalizing. Many psychoanalysts still adhere to a motivational theory, which emphasizes that “relational needs” become thwarted by libidinal and aggressive drives. Furthermore, the mental maturation of the 5- to 6-year-old child depends on a successful solution of the oedipal conflict, whereby the perspectives of the father/mother/society become internalized, and not being a result of the child’s increased capacity for explicit mentalization, whereby it can look upon itself from “the outside,” filtered through societal norms.

As outlined above, the TAM theory is well equipped for constructive dialogs with the FFM model, social-cognitive theories of personality and psychoanalysis. It borrows crucial validated concepts (e.g., conscientiousness) while also offering a new synthesis (e.g., how temperament, attachment, and mentalizing are interwoven), together with a “translation” of established concepts such as “attachment” and factors of the FFM to more evolutionary and neurobiological and motivational anchored constructs, e.g., in terms of primary emotions. We will maintain that this new synthesis, or integration, constitutes a personality theory that is more comprehensive and valid than its forerunners and thereby provides a larger explanatory power. It may serve a host of purposes, as indicated in the following paragraphs. As mentioned in the introduction, we are particularly concerned with the need of grounding personality disorders in a valid theory of personality, a theme to which we now turn.

### Relevance for Understanding Personality Disorders

By making use of the TAM scheme which we have outlined above, as well as recent research in this area that demonstrates the correlations between primary emotions and personality disorder criteria (Karterud et al., 2016), we will illustrate the explanatory power of the TAM theory by describing how different personality disorders can be interpreted as different constellations of various aspects of temperament, attachment, and mentalizing profiles. To conserve space, we limit this to a description of borderline, narcissistic, and avoidant personality disorder, as well as to some remarks regarding the DSM-5 Section III alternative model.

#### Borderline Personality Disorder (BPD)

In contrast to some other theoretical formulations (e.g., Linehan, 1993), we do not regard BPD primarily as an emotion regulation disorder but rather as a disorder of personality in the sense that *all of the three major components are affected*: temperament, attachment, and mentalizing capability. The temperament or primary emotional system liability of BPD concerns impulsivity as well as low threshold, high intensity, and regulation problems, particularly of the primary emotions of RAGE and SEPARATION DISTRESS (Karterud et al., 2016). Separation distress accounts for the profound dysphoria of being left alone and the desperate attempts at avoiding real or imagined abandonment. The tragedy for BPD patients is that their proclivity for rage reactions enhances the risk of being left alone. Moreover, their difficult temperament, notably widespread irritability and proneness to intense rage reactions, may also play a major role in accounting for their very poor functioning in society (Hastrup et al., 2018).

An intense temperament is not enough for a borderline condition. Additionally, there must also be an insecure attachment pattern – prototypically of an overinvolved or disorganized type (Karterud et al., 2017). However, all types of insecure attachment may be encountered in BPD patients. Considering that most of the empirically supported treatments for BPD contain group therapy (Storebø et al., 2018), it is particularly important for practitioners to become aware of a disorganized pattern, as especially patients with this pattern will have great problems in group therapy (Morken et al., 2014). Realizing that a disorganized attachment pattern is operative makes it easier to understand the patients’ reluctance to group involvement and their need for time and patience in order to approach fellow patients.

The mentalizing problems of BPD patients account for their poor sense of self. The problems are at least twofold. First, there is the generally lowered capability of mentalizing, which makes the person liable to misunderstanding of others and oneself and thereby exploitable and exploiting. In addition, there is the liability for gross breakdowns of mentalizing abilities and the risk for (self-) destructive acting out. Both deficits affect the capability for self-understanding and experience of identity and agency. The deficits can be traced back to failures in the formative parent-child interaction, whereby the child’s subjective experiences are victims of faulty mirroring and distorting intersubjective transaction (Fonagy et al., 2005; Bo et al., 2017). Moreover, such failures in the parent-child interaction may also lead to the severe epistemic mistrust, which is found in BPD (Fonagy and Allison, 2014; Bo et al., 2017). Briefly, this theoretical reasoning highlights that one of the most devastating developmental consequences of impoverished, insecure, or traumatic parenting is that it leaves the child in a state of epistemic mistrust. Attachment trauma undermines the child’s capacity for trust in others and hence the individual’s ability to learn from others.

#### Narcissistic Personality Disorder (NPD)

NPD represents quite a different combination of temperament, attachment, and mentalizing. Concerning temperament, NPD is substantially associated with social dominance and (negatively) with CARE (low empathy) (Karterud et al., 2016). This finding is in accordance with clinical observations, social-psychological research, and other test results (Ronningstam, 2005; Karterud, 2010; van der Westhuizen and Solms, 2015). Add a narcissistic individual to a group (e.g., therapy group), he/she will go for dominance and leadership and not so much for the care of others or being particularly empathic. What about their attachment pattern? The number of relevant studies is low, but they point to a dismissive attachment pattern being the prototype (Rosenstein and Horowitz, 1996). Narcissistic individuals will often know a lot of people but have few, if any, intimate relations. They seldom let others come close enough to experience their (self-loathed) weak sides. A low self-compassion corresponds with low empathy for the suffering of others. And their mentalizing difficulties? In accordance with a dismissive attachment pattern (Crittenden, 2003), narcissistic individuals tend to be cognitively oriented, more than affective. Their cognition tends to be overly rationalistic and not so much informed by fine-grained emotional attunement. Their understanding of others will therefore tend to be superficial (pseudomentalizing), and they will tend to over-evaluate themselves since they lack any true emotional awareness of their weak sides. The tragedy for persons with NPD is that in general, power and status are granted by others to people whom they feel care about them and will do good for them (Keltner, 2016). This requires good social skills, including sound attachment abilities, empathy/CARE, and mentalizing capacities – none of which the prototypical person with NPD does possess. Hence, their striving for status and power, more often than not are doomed to fail sooner or later, and they risk becoming recurrently depressed and commit suicide (Coleman et al., 2017).

#### Avoidant Personality Disorder (AvPD)

AvPD is, according to TAM theory, characterized by a temperamental disposition of low threshold for FEAR and high thresholds for SEEKING, PLAY, and RAGE (Karterud et al., 2016). It is unresolved whether fear inhibits seeking, play, and rage, or if there is a true low-keyed temperamental disposition for these last three primary emotions. The predominance of fear has been observed repeatedly among young inhibited children and shown to persist into adulthood (Schwartz et al., 2003; Kagan et al., 2007). Fear is accompanied by avoidant behavior, which is a predominant feature of AvPD. There are divergent findings with respect to attachment patterns among subjects with AvPD (Eikenaes et al., 2016). The mentalizing problems of AvPD subjects fall into two major categories. First, there is the devaluation of own self. AvPD subjects tend to look upon themselves as inferior, less attractive, and worthless. In cognitive theory, this feature is considered a prime example of an early maladaptive self-devaluating schema. Mentalization theory conceptualizes this as implicit (automatic) mentalizing deficits. In addition, AvPD subjects also harbor difficulties in understanding the mind of others (Dimaggio et al., 2007; Lampe and Malhi, 2018). Another major mentalizing deficit is their poor affect consciousness (McCullough et al., 2003; Solbakken et al., 2011; Gordon-King et al., 2019).

#### DSM-5 Alternative Model

Although the revision process of the chapter on personality disorders in DSM-IV did not succeed in full agreement of an alternative model (American Psychiatric Association, 2013), the discussions revealed a broad agreement on the principle of dimensionality and a conceptual delineation of level of personality together with trait characteristics. The TAM theory is compatible with such a view. Level of functioning in the alternative model in DSM-5 refers to differences between individuals with respect to identity, self-direction, empathy, and intimacy (Bender et al., 2011). These areas are covered by the TAM theory.

The personality trait system in the alternative model includes the 25 facets subsumed under the following five higher-order domains: negative affectivity (vs. emotional stability), detachment (vs. extraversion), antagonism (vs. agreeableness), disinhibition (vs. conscientiousness), and psychoticism (vs. lucidity) (Krueger et al., 2011; American Psychiatric Association, 2013). By and large, the five domains represent maladaptive variants of the FFM. But how does the DSM-5 trait model align with the primary emotions? To the best of our knowledge, there have not been conducted any empirical studies to address this. However, we would hypothesize, based on content analysis and studies on associations between primary emotions and the FFM (e.g., Montag and Panksepp, 2017), that FEAR and ANGER are associated with the domain of negative affectivity. Detachment may capture pathological low levels of PLAY and high levels of SADNESS. Moreover, LUST may also fall within this domain and more specifically the facet of intimacy avoidance. Antagonism may be negatively associated with CARE. Though SEEKING is associated with openness in the FFM, we are not sure that this would be the case regarding the domain of psychoticism. Future studies ought to examine the links between primary emotions and DSM-5 trait domains.

Although the ICD revision process was completed in 2018 with the launch of ICD-11, the future of the DSM-5 section for personality disorder is still open. Does the TAM theory carry any implications besides a modification of the trait domains? As we have suggested in previous paragraphs on borderline, narcissistic, and avoidant PDs, the TAM approach seems to carry a high explanatory power for *personality types.* The personality type approach is controversial since it carries with it the danger of being misinterpreted as “disorders” in a strong biological sense, more like “diseases.” However, a sounder interpretation of personality types is that they represent personality prototypes (which real persons might more or less be resemble) (Johansen et al., 2004). We would suggest that future DSM revision process not only fulfills the promising work with the levels of personality functioning but also does a more conservative revision of the traditional personality (disorder) types, which will preserve the historical continuity with DSM-IV. Furthermore, we will suggest that the criteria for the different personality disorder prototypes adhere more closely to temperament characteristics, attachment styles, and mentalizing difficulties.

### Assessing the TAM Components

In the introduction, we stated that requirements for a valid theory were that its core components could be measurable, so now we will indicate how the core constituents of personality can be assessed for clinical and research purposes. Primary emotions can be measured by the Affective Neuroscience Personality Scales (ANPS) (Davis et al., 2003; Pedersen et al., 2014; Montag and Panksepp, 2017). ANPS is a self-report questionnaire with acceptable psychometric properties, which, however, might profit from a psychometric refinement. The main problems with ANPS are some overlap between the care and separation distress items, lack of a lust scale, and a limited dominance scale (Pedersen et al., 2014). However, van der Westhuizen and Solms (2015) have provided promising new scales for lust and social dominance, and if their findings can be replicated, the official ANPS scale should be modified accordingly. Furthermore, the ANPS could be supplemented with the Conscientiousness scale from NEO-PI-R (McCrae et al., 2005). Such an extended ANPS might become a useful instrument for assessing human temperament.

Generally speaking, assessing attachment patterns have for long been a problem for clinical psychology. The Adult Attachment Interview (AAI) is a research tool, which is too time consuming for clinical work (Hesse, 2008). Based on our experience from teaching doctors and psychologists in Scandinavia, we have realized that most of them are reasonably skilled in assessment of personality pathology, but only rarely assess the attachment patterns of their patients. Though assessment of attachment orientation preferably requires a semi-structured interview, this is often only realistic in research projects, whereas busy clinicians need brief instruments. In our opinion, the most useful tool available for routine clinical practice is probably the Experiences in Close Relationship Questionnaire (Fraley et al., 2000), which measures the avoidance and anxiety dimensions of attachment.

The level of mentalizing ability is a major component of self-consciousness and thus a most important factor to consider when planning treatment (Bateman and Fonagy, 2016). Assessing the multifaceted construct of mentalizing is, however, challenging (Kongerslev et al., 2015), notably for clinicians, though a number of psychometrically sound assessment tools are becoming available (Luyten et al., 2012). The Reflective Functioning Scale (Fonagy et al., 1998), which was the original empirical measure of mentalizing, relies on the AAI, thus primarily being a research tool, and only captures the construct of mentalizing at a general level. Recently, the Reflective Functioning Questionnaire (RFQ) has been developed and validated for use with both adolescents and adults (Ha et al., 2013; Fonagy et al., 2016). This self-report inventory is promising for routine use in clinical practice. Moreover, there are several indirect means of structured assessments, which can be used to gauge the level of mentalizing, including, e.g., the Global Assessment of Functioning (GAF) or the Level of Personality Functioning Scale (Bender et al., 2011). Narratives of mentalizing deficits might also be obtained from the Interview on Mentalizing Failures (Karterud et al., 2017).

### Implications for Self-Understanding

In the introduction, we stated that a valid and useful theory of personality should carry with it implications for commonsense self-understanding. Every individual will ask him/herself, in some way or the other, the questions “who am I?” and “what am I like?” How should the individual be guided in this respect? How can this broad existential question be parceled into manageable parts? The TAM theory might suggest the following route: start with your emotions. Have you access to all of them? If not, which come easily, and which are hard to be aware of? Are you in control of your emotions, or do they control you? Why is it so? Proceed with your attachment pattern. Is it secure or insecure? Overinvolved or dismissive? Are you satisfied with it? Should you do anything to improve your emotional awareness and attachment pattern? Next, how is your mentalizing capacity and style? Is it restricted in some way? How do you proceed in constructing your autobiographical memory and narrative identity? Which cultural resources do you make use of? How is your personal narrative embedded in the story of your family, work, and socio-political situation? What kind of dialogs are you involved in with respect to these matters? Who do you talk to and how? It follows also from the TAM theory that personality development is a never-ending story and a moral obligation. To a large extent, individuals are responsible for their personality. One should take the inscription on the temple of Delphi seriously: “Know thyself.” One should consider it a virtue to continuously refine one’s personality.

### Implications for Social Psychology

The TAM theory of personality carries some normative views, in the sense that certain personality configurations are considered as “better” than others. “Better” means that they are associated with more personal happiness, health, and wellbeing than others and that they are better for society as a whole (Lyons-Ruth et al., 2006; Roberts et al., 2007; Baumeister and Vonasch, 2012; Reizer et al., 2013; Beeney et al., 2017; Bo and Kongerslev, 2017). Examples: It is better to be aware and tolerant of one’s emotions, than being alexithymic. Or: High emotional consciousness is better than low emotional consciousness. It is better to be in control of one’s emotions than being dysregulated and driven by one’s emotions. Furthermore, it is better to have a secure attachment pattern than an insecure. It is better for the person, his/her partner, children, and family. It is also better for society at large. And finally, one is better off with a high capacity for mentalizing than with a low. A high mentalizing capacity opens for profound, sincere, and interesting relations with other people, while a low capacity is ripe with frequent misunderstandings, empathy defects, hurting others, being hurt oneself, lack of abilities to resolve intersubjective disputes, etc. Accordingly, the TAM theory carries wide implications for childrearing practices, kindergarten politics, and school ethos, curricula and courses, family politics, family therapies, human rights, and media politics. For example, it is hard to mentalize properly when your (re-)sources are limited by a dictatorial and/or religious fundamentalist distortion of public discourse.

It follows from the relational essence of all three personality constituents that individuals are embedded in socio-cultural practices and that their personality characteristics are not written in stones. There is thus a personality/family/group/culture/politics dialectics. The seemingly “moralistic” stance of the previous paragraph that places strong burdens on the shoulders of the individual has to be supplemented by a likewise strong responsibility of social agencies, e.g., that they provide support for sound personality development and prevent personality disintegration. Worst case scenarios are state disintegration with breakdown of civic order with resulting chaos, violence, terrorism, and lack of trustworthy authorities. There are indeed evidence suggesting links between destructive social and societal conditions and poor personality functioning or conversely between favorable social and societal conditions and adaptive personality functioning (Chapman et al., 2010; Ungar, 2015; Svendsen and Svendsen, 2016; Fukuyama, 2018).

### Implications for Treatment

The TAM theory has implications for treatment across different psychotherapeutic schools and modalities (see e.g., Karterud et al., 2019). It does not prescribe any particular method. However, it is most helpful in assessing the ailment and may assist in pointing to the best possible remedy. How is the overall personality structure and functioning? For example, in the terms of levels of personality functioning as described in the DSM-5 Section III. Which temperamental factors create problems for the individual? How should they be addressed? Can the emotions be approached with a short-term and direct technique, as in emotion-focused therapy? How is the specific attachment style of this particular patient? In which way does it create problems? Is there, e.g., any overinvolvement with poor boundaries toward significant others? Is it described in the case formulation? (Karterud and Kongerslev, 2019). Is there any motivation for change in this respect? How does the attachment pattern reveal itself in the transference? Does the therapist work with it in the transference? Where does the mentalizing problems reside? In which personality realms and which situations does the patient display poor mentalizing and where is it good enough? Does it predominately concern the self or others, emotions or cognition? Are the mentalizing problems delineated, e.g., are there some few dysfunctional cognitive schemata, which may indicate cognitive therapy? Or are they pervasive and call for more complex and long-term interventions, like mentalization-based treatment (MBT)? The questions and challenges are numerous. The TAM theory might help to organize thinking about these questions (Karterud and Kongerslev, 2019).

### Limitations

As the theme of this article is broad in scope and we attempt to integrate such a wide range of theoretical and empirical traditions, we assume that many readers who are engaged in the discourse of personality and its disorders will miss a more varied and detailed account of important aspects. For example, we have not commented on the developmental dynamics throughout the lifespan and the temperament research tradition of Posner and Rothbart (1998). Nor have we discussed issues of impulsivity and effortful control (MacDonald, 2008). Also, we have used the term mentalizing as a common term without discussing important subtle nuances of closely related terms such as social cognition and metacognition (for a conceptual clarification of this, see e.g., Dimaggio et al., 2015). We have referred to the basics of mentalization theory but only in passing to its mature elaborations of broader narrations, e.g., its capacity for constructing a narrative identity and thereby a narrative unity in life (McLean et al., 2007; McAdams, 2018). We would particularly have liked to discuss more in depth the dialectics of the core self and self-consciousness/mentalization, e.g., the former carrying with it the stability of personality traits (sameness), while mentalization anchors the traits in a culturally appropriate narrative discourse and represents a modicum of freedom and transcendence. Moreover, we wish to express our debt to influential authors like Lichtenberg et al. (1992) and Liotti and Gilbert (2010) who have been pioneers in the effort to integrate evolutionary and psychodynamic theories. We will also acknowledge the more recent contributions by Dimaggio and coworkers (e.g., Dimaggio, 2015; Dimaggio et al., 2015; Dimaggio and Lysaker, 2018). The strength of these authors’ thinking is their highly clinically relevant way of thinking about the evolutionary roles of motivational systems, attachment disturbances, mentalizing and social mentalities, and their consequences for functioning. Lichtenberg, Liotti, and Gilbert share similar views on evolutionary grounded “motivational systems” (e.g., for attachment, sexual pair-bonding, alliance building and cooperation, and dominance-submission). However, we will argue that there is much to gain in explanatory power if these motivational systems are translated into primary emotional systems. For example, as we have explained in a preceding paragraph, “the attachment system” can be deconstructed to an interplay of FEAR, SEPARATION DISTRESS, CARE, and SEEKING, with their different neurotransmitters, feeling states, behavioral tendencies, etc. Overall, we have focused on the major constituents of personality and not so much upon the details of the fabric of the adult mind, which would include, e.g., self- and interpersonal schemas, motivation and goals, subjectivity and intentionality, defense mechanisms, identity, and ethics and moral.

## Conclusion

Personality theory has to be redefined when new empirical and conceptual developments accumulate. Such developments have taken place within affective neuroscience, evolution, and social cognition. In this article, we have outlined how we now may conceive a theoretical web that links evolution of temperament by natural selection, to lived attachment experiences, and finally to internalization of cultural achievements that favor sophisticated self-understanding. Individuals differ in these regards, and they might be assessed and ascribed different personality profiles, which have strong predictive validity for successful versus unsuccessful functioning of their life course. In the theoretical web, we have outlined transcends traditional contradictions between natural sciences and hermeneutics. We have suggested a theory where the crucial elements build on each other, including the final step of self-consciousness, i.e., the ability to interpret others and self. We admit that many aspects of the theory should be elaborated more in detail. That is a challenge for the future.

## Author Contributions

SK drafted the first version of this manuscript. MK worked over this version and revised it critically as well as wrote the introduction.

### Conflict of Interest Statement

The authors declare that the research was conducted in the absence of any commercial or financial relationships that could be construed as a potential conflict of interest.
